# The effects of perceived teacher support and growth language mindset on learner well-being in AI-integrated environment: the mediating role of generative AI attitude

**DOI:** 10.3389/fpsyg.2025.1660462

**Published:** 2025-09-16

**Authors:** Yong Pan, Gengchun Li

**Affiliations:** School of Foreign Languages, Taizhou University, Taizhou, China

**Keywords:** perceived teacher support, growth language mindset, generative AI, well-being, Chinese EFL learners

## Abstract

**Introduction:**

With continuing advancements in AI technologies, the landscape of English as a foreign language (EFL) teaching and second language acquisition (SLA) is undergoing transformative changes, with learners' emotions in AI-integrated environments capturing more and more research attention. However, a dearth of studies has paid attention to learner well-being in such environments and its influencing factors.

**Methods:**

Based on previous research on perceived teacher support (PTS) and growth language mindset (GLM) in EFL educational contexts, the present study explored the possible direct effects of PTS and GLM and the mediating effect of generative AI attitude on learner well-being among a sample of Chinese university EFL learners (*N* = 486), using a questionnaire survey, and utilizing statistical procedures such as correlation analysis, regression analysis, and mediation analysis.

**Results:**

The results indicated that: (1) both PTS and GLM significantly and positively predicted generative AI attitude and learner wellbeing; (2) generative AI attitude significantly and positively predicted learner well-being; and (3) generative AI attitude played a partial and positive mediating role in the relationships between PTS/GLM and learner wellbeing.

**Discussion:**

The research findings are expected to provide practical insights into fostering university EFL learners' well-being in AI-integrated environments.

## Introduction

Artificial intelligence (AI) is a broad term covering various technologies and methods, not just one specific technology (Guo and Wang, 2025), including natural language processing, machine learning, and neural network architectures. In a broad sense, AI refers to computer systems performing cognitive tasks traditionally associated with human intelligence, such as learning, reasoning, problem-solving, perception and language understanding (Wang, 2025). Generative AI, specifically, refers to sophisticated AI systems which employ machine learning algorithms to create original content, including text, images, and audio, driven by advancements in deep learning and neural network architectures (Huang et al., 2024).

In the educational domain, AI holds profound implications due to its capacity to optimize teaching activities, enable personalized instruction, and enhance learning outcomes (Wu et al., 2024). Notably, the advancements in AI have significantly transformed second language acquisition (SLA), profoundly influencing learners' linguistic outcomes and the dynamics of their learning processes (Huang et al., 2024). Consequently, it has given rise to a new line of research, i.e., AI-assisted second/foreign language teaching and learning.

In language learning, an array of emotions, both positive and negative, exert a profound influence on learner's success, ranging from enjoyment and hope to anxiety and boredom (Wang and Wang, 2025); for instance, foreign language classroom anxiety can serve as a major barrier, hindering learners' willingness to participate in class activities and take risks, whereas enjoyment is often positively correlated with motivation, persistence, and ultimate achievement (Wang, 2025). As relatively nascent technologies, the integration of AI into EFL teaching and learning raises a significant question worthy of in-depth exploration: What emotional experiences may it engender among EFL learners? Therefore, in recent years, EFL learners' emotions in AI-integrated environments have received more and more attention. Existing studies suggest that the integration of AI technologies may elicit both positive and negative emotions among EFL students (Guo and Wang, 2025; Wang et al., 2023; Yang and Zhao, 2024). For instance, the study by Guo and Wang (2025) found that in AI-enhanced instructional settings, learners' emotional experiences were rich and dynamic, encompassing 11 types of positive and negative emotions, respectively, with the former occurring more frequently (149 vs. 71 coded instances). Yang and Zhao (2024) also found that Chinese EFL learners exhibited a wide range of both positive and negative emotional experiences in AI-based L2 education.

However, among the existing studies on emotions in AI-integrated environments, EFL learners' well-being has received much less attention (e.g., Huang et al., 2024; Linh et al., 2022; Zong and Yang, 2025). Well-being is an important construct in positive psychology that eludes a universally accepted definition due to its intricate nature and individual variations in its perception (Li, 2025). According to Tu and Shi's (2024) conceptualization, well-being refers to a holistic psycho-emotional state encompassing an individual's psychological, emotional, spiritual, intellectual, physical, and social wellness. As a positive health-related outcome (Wang Y. et al., 2021), well-being facilitates personal growth and flourishing (Seligman and Csikszentmihalyi, 2000). According to Seligman's PERMA model (Seligman, 2011), well-being emerges from the common development of five components or pillars: positive emotions, engagement, relationships, meaning, and accomplishment. Among them, “positive emotions” can be understood as hedonic-like affections associated with pleasure and happiness; “engagement” refers to being fully concentrated on a pleasant and interesting activity; “relationship” denotes positive relationships that are related to the quality and availability of social bonds; “meaning” entails having a clear sense of purpose in life and finding meaning in what one does; and “accomplishment” signifies the sense of fulfillment and self-actualization that individuals obtain when achieving the goals that are important to them (Li, 2025, p. 4).

As a psycho-emotional construct encompassing both positive emotions and optimal psychological functioning (Li, 2025), well-being serves as a foundation for effective learning and overall life satisfaction in language acquisition (Huang et al., 2024). Empirical findings indicate that EFL students with higher levels of well-being demonstrate superior interpersonal communication skills, along with greater self-efficacy, self-esteem, rapport, emotional regulation, and engagement (Tu and Shi, 2024). Moreover, well-being has been argued to significantly predict academic engagement and enhance EFL learning outcomes (Xu and Feng, 2025).

Nevertheless, in AI-integrated environments, EFL learners may face dual challenges that constrain and influence their perceptions of well-being. The first challenge stems from AI technologies themselves. For EFL learners with limited exposure to AI technologies, incorporating AI-powered tools or systems into learning may evoke negative emotional responses, such as anxiety and frustration, especially when students perceive inadequate support or excessive complexity (Yang and Rui, 2025). Relatedly, if AI tools or systems were originally designed without sufficient consideration of EFL learners, they may inadvertently exacerbate feelings of alienation or anxiety (Yang and Rui, 2025). The second challenge arises from the inherent difficulties of SLA, which arguably is a long-term and arduous process, during which learners frequently encounter frustrations and challenges, leading to negative emotions such as anxiety, shame, boredom and helplessness (see Li and Xu, 2019 for more negative classroom emotions).

Given these challenges, social support, particularly teacher support in school settings, emerges as a critical buffer against academic and psychological difficulties (Malecki and Elliott, 1999). Teacher support has been shown to directly mitigate negative emotions (e.g., anxiety, boredom) while fostering positive emotions, including classroom enjoyment (e.g., Liu et al., 2025; Zhao and Yang, 2022). This, in turn, contributes to a more engaging and motivating L2 learning environment. Consequently, understanding and addressing EFL students' perceptions of teacher support is essential for cultivating a supportive learning atmosphere conducive to academic success and personal growth (Derakhshan et al., 2025). However, research on PTS in AI-integrated EFL contexts remains notably scarce, limiting our understanding of the relationship between teacher support and learner well-being in such environments. Furthermore, scholars have highlighted that learners' language mindsets are closely associated with their language learning behaviors and attitudes (Lou and Noels, 2016) and may exert a significant influence on foreign language (FL) emotions (King et al., 2012). Indeed, empirical studies to date have demonstrated that a growth language mindset (GLM) negatively predicts FL anxiety (e.g., Lou and Noels, 2017; Zarrinabadi et al., 2022) and boredom (Wang H. et al., 2021), while positively predicting enjoyment (Wang H. et al., 2021; Zarrinabadi et al., 2022) and pride (Wang H. et al., 2021). It has been argued that individuals with a GLM perceive SLA as an opportunity for self-development and skill enhancement, and therefore they demonstrate greater willingness to embrace challenges and exhibit resilience in the face of failures (Rong and Liu, 2024). Given these findings, GLM may represent another critical variable influencing learners' well-being. However, research on GLM among EFL students in AI-integrated environments remains inadequate, leaving its potential influence on learner well-being in such contexts under-explored.

Given the observed correlations between PTS/GLM with a range of emotions in SLA, coupled with the research gaps identified earlier, this study focuses on Chinese EFL learners to specifically investigate the influence of PTS and GLM on learner well-being, and the potential mediating role of generative AI attitude in this relationship, by gleaning insights from multiples theories in social and educational psychology, including Self-Determination Theory (Deci and Ryan, 2000; Ryan and Deci, 2000), Broaden-and-Build Theory (Fredrickson, 2001), and Achievement Goal Theory (Elliot and Church, 1997). By elucidating these mechanisms, the study aims to deepen our understanding of the key influencing factors on learner well-being in generative AI-integrated environments. The findings are expected to provide an empirical foundation for targeted pedagogical interventions to enhance EFL learners' well-being in an era of generative AI.

## Literature review

### Theoretical foundation

Three canonical theories in social and educational psychology informed both the design of this study and interpretation of its results, including Self-Determination Theory (Deci and Ryan, 2000; Ryan and Deci, 2000), Broaden-and-Build Theory (Fredrickson, 2001), and Achievement Goal Theory (Elliot and Church, 1997).

Self-Determination Theory (SDT) offers insights that are instrumental in elucidating the role of teacher support in students' well-being. According to SDT, individuals universally possess innate psychological needs for autonomy, competence, and relatedness, and social environments can facilitate motivation and behavioral development by supporting and satisfying these fundamental needs (Zhang and Wang, 2023). Specifically, within classroom settings, teacher support, as a critical component of social support, enables students to experience heightened autonomy and relatedness, thereby internalizing motivational behaviors and promoting more active engagement in learning activities (Niemiec and Ryan, 2009). The theory further posits that supportive interpersonal relationships that fulfill students' basic psychological needs can shift learners' focus from external evaluation to internal developmental factors such as personal growth. This shift enhances their environmental adaptive capacity and emotional regulation, thereby fostering greater learner well-being (Hu and Zhu, 2025). Therefore, it is expected in this study that timely and sufficient teacher support will lead to an enhancement of learner well-being.

Broaden-and-Build Theory (BBT) holds certain promise for explaining the effect of generative AI acceptance on learners' well-being. According to BBT, positive emotions facilitate the broadening of attention and mind, as well as the expansion of behavioral repertoires. When these short-term effects accumulate over time, they promote the building of physiological, psychological, and social resources that foster personal development. The accumulation of such resources and personal development, in turn, reinforce the generation of further positive emotions. Moreover, positive emotions can buffer against the detrimental effects of negative emotions, thereby fostering resilience and enhancing well-being (Li et al., 2024). Accordingly, positive attitudes toward technological acceptance, which fall within the broad category of positive emotions, are hypothesized in this study as another factor closed linked to learner well-being.

The Achievement Goal Theory (AGT) provides insights that contribute to an understanding of the role of growth language mindset in learner well-being. One of the core concepts of this theory is achievement goal orientations, which have to do with the reasons behind learners' engagement in certain academic activities. From a social-cognitive perspective, Dweck and Leggett (1988) first proposed two distinct constructs of goal orientations: mastery goal orientation and performance goal orientation. Subsequently, Elliot and Church (1997) further subdivided the performance goal orientation into performance-approach orientation and performance-avoidance orientation, thereby establishing a trichotomous model of achievement goals. It is posited that learners with a mastery goal orientation tend to focus on gaining an in-depth understanding of knowledge, acquiring new knowledge, and achieving self-improvement through the acquisition of new skills and competence. In contrast, learners with a performance-approach orientation are inclined to demonstrate their knowledge and abilities to obtain positive evaluations; and those with a performance-avoidance orientation strive to avoid negative evaluations resulting from insufficient ability or poor performance (Zhang and Zhang, 2024). It is posited by researchers that EFL learners with a growth language mindset predominantly adopt a mastery goal orientation (e.g., Camacho et al., 2022; Lou and Noels, 2017), and therefore they would concentrate on improving their language skills and competence, without excessive concern over performance outcomes or external evaluations, and as a result, they would reap more well-being than those with performance goal orientations.

### PTS and learner well-being

As a multidimensional construct, teacher support (TS) has been defined differently from two theoretical perspectives (Zhao and Yang, 2022). According to SDT, TS consists of three dimensions: autonomy support, involvement support, and structure support (Lei et al., 2018). Autonomy support refers to teachers respecting students' choices, encouraging self-directed decision-making, and minimizing controlling behaviors to foster intrinsic motivation. Involvement support involves teachers establishing emotional connections and showing care, helping students feel a sense of belonging and value to enhance their engagement in learning. Structure support entails teachers providing clear expectations, guidance, and feedback to help students establish goals and rules, thereby strengthening their perceived competence. From the perspective of social support, TS can be defined as the informational, instrumental, appraisal, and emotional support that teachers provide to students (Malecki and Demary, 2002). Based on the social support model, Liu and Li (2023) developed and validated the *Students' Perceived EFL Teacher Support Scale*. They found that EFL teacher support is a three-dimensional construct, consisting of academic support, instrumental support, and emotional support. Specifically, academic support refers to EFL teachers imparting English language knowledge and offering constructive feedback to students; instrumental support includes providing learning materials (e.g., textbooks and online resources) and dedicating extracurricular time to students; emotional support encompasses teachers' trust, empathy, care, and concern for students (Li et al., 2023; Liu and Li, 2023). Additionally, TS can be categorized into provided teacher support and perceived teacher support, with the latter being more predictive (Gu et al., 2023). Of particular note is that the nature and internal structure of TS vary across disciplines, and students from different academic fields may require different types of support (Liu and Li, 2023). Given the EFL learning context of this study, we adopt Liu and Li's (2023) conceptualization of TS and its validated scale, and define PTS as the support that learners perceive from their EFL teachers during the learning process, including academic, instrumental, and emotional support.

Empirical studies on EFL teacher support has primarily focused on its influence on students' academic engagement and achievement, as well as its relationship with students' psychological or emotional factors (see Li et al., 2023; Liu and Li, 2023). For instance, Tao et al. (2022), through an analysis of 71 empirical studies, revealed that TS had the most significant impact on high school students' academic achievement and course performance. Yang et al. (2024) explored the relationships among PTS, grit, and L2 willingness to communicate, as well as the mediating role of FL enjoyment. The results indicated that TS was strongly correlated with grit (*r* = 0.647) and FL enjoyment (*r* = 0.671), and that TS positively predicted FL enjoyment (β = 0.277, *p* < 0.001). Zhao and Yang (2022) examined the relationships among students' PTS, enjoyment, boredom, and academic engagement in Chinese EFL context. The findings demonstrated moderate to high correlations among PTS, enjoyment, boredom, and academic engagement, and that enjoyment and boredom jointly mediated the relationship between PTS and academic engagement. Thus, in EFL settings, TS serves as a crucial contextual factor and may represent an important resource for students to enhance academic engagement, improve academic achievement, and foster positive emotions during the learning process (Zhao and Yang, 2022).

However, research on the role of TS in relation to learner well-being in AI-integrated environments remains scarce. In a related study, Huang et al. (2024) investigated the effects of generative AI acceptance, perceived teachers' enthusiasm, and self-efficacy on the well-being of 613 university EFL learners. The results revealed that perceived teachers' enthusiasm positively predicted EFL learners' self-efficacy but had no predictive effect on their well-being; instead, the relationship between perceived teachers' enthusiasm and well-being was mediated by receptive skill self-efficacy. It thus appears from this study that perceived teachers' enthusiasm needs to work with other variable(s) in order for the levels of well-being to improve. That being said, it remains unclear whether other teacher factors such as perceived teacher support will be able to enhance learner well-being more significantly. Actually, the concept of perceived teachers' enthusiasm differs considerably from that of perceived teacher support. According to Huang et al. (2024), perceived teachers' enthusiasm refers to students' perceptions of their teachers' energetic, passionate, and positive teaching practices, including the teacher's excitement about the subject matter and genuine interest in student learning, which largely differs from the construct of PTS as introduced earlier. Therefore, it is interesting to know whether PTS directly influences EFL learners' well-being in AI-integrated environments.

### GLM and learner well-being

Language mindsets refer to learners' beliefs about the malleability of their language abilities and aptitude (Lou and Noels, 2017). They can be categorized into two types: growth language mindset (GLM) and fixed language mindset (FLM). The idea of GLM entails that general language abilities and L2 aptitude can be developed through effort and are not constrained by innate talent or starting age of L2 learning. In contrast, the concept of FLM assumes that language abilities and L2 aptitude are innate, remain stable throughout one's lifespan, and cannot be significantly improved through effort (Li et al., 2023). Learners with a GLM tend to seek challenging tasks and attribute failures to controllable factors such as insufficient effort or strategy use, thereby increasing subsequent effort; conversely, those with a FLM tend to avoid challenges and attribute failures to uncontrollable factors such as lack of innate talent, leading to reduced effort (Zhang and Jiang, 2024). Consequently, scholars have suggested that learners' language mindsets are associated with their language learning behaviors and attitudes (Lou and Noels, 2016) and may significantly influence FL emotions (King et al., 2012).

Empirical studies have indeed confirmed the close relationship between language mindsets and FL emotions. For example, Wei and Chen (2022) found that among 622 Chinese non-English major freshmen, GLM was significantly positively correlated with enjoyment and self-efficacy but negatively correlated with anxiety. Conversely, FLM was significantly negatively correlated with enjoyment and self-efficacy but positively correlated with anxiety. Furthermore, GLM significantly and positively predicted enjoyment, whereas FLM negatively predicted enjoyment and positively predicted anxiety. Wang H. et al. (2021) found that GLM positively predicted enjoyment and pride but negatively predicted boredom. Zarrinabadi et al. (2022) demonstrated that GLM positively predicted enjoyment and negatively predicted anxiety, both through adaptability, while FLM directly and positively predicted anxiety and negatively predicted enjoyment through adaptability.

These findings suggest that GLM may be positively associated with EFL learners' positive emotions and negatively related to their negative emotions. Since positive emotions constitute an essential element of well-being (Seligman, 2011) and demonstrate significant positive correlations with other elements of well-being (Li, 2025), it is reasonable to hypothesize that GLM may be closely linked to well-being. However, few studies have explored the relationship between language mindsets and well-being, and even fewer have examined how GLM relates to EFL learners' well-being in AI-integrated learning environments. This gap in the literature warrants further investigation.

### Generative AI attitude and learner well-being

Attitude refers to an individual's relatively stable psychological disposition toward specific entities, including people, ideas, affect, or events (Wang et al., 2024). Based on the tripartite structure of attitude (i.e., affect, behavior and cognition), Wang et al. (2024) defines generative AI attitude in the EFL context as learners' value judgments regarding the application of generative AI technologies in English learning processes, which encompasses three dimensions: affective, behavioral, and cognitive. The affective dimension refers to the emotional experiences of EFL learners with generative AI tools or systems. The behavioral dimension denotes the reaction tendency or behavioral readiness of EFL learners toward generative AI tools or systems. The cognitive dimension involves learners' evaluative beliefs about generative AI, including their perception, trust, skepticism, as well as approval or disapproval of such tools or systems (Wang et al., 2024, p. 31).

Attitudes toward generative AI are generally regarded as a pivotal component within the Technology Acceptance Model (TAM), originally proposed by Davis (1989) in the field of computer technology based on the Theory of Reasoned Action, to explain and predict user acceptance of information technology. TAM posits that users' behavioral intention to adopt a technology is determined by three key factors: perceived usefulness, perceived ease of use, and attitude toward use (Davis, 1989). Within this model, behavioral intention constitutes the primary outcome, as it directly predicts actual usage behavior (Davis, 1989; Teo et al., 2017). It is held by this model that both perceived usefulness and perceived ease of use will exert an influence on user's attitude toward use, and moreover, perceived usefulness and attitude toward use will have an impact on behavioral intention (Wu et al., 2024).

Previous studies have shown that positive attitude toward AI serves as a significant predictor of behavioral intentions to adopt AI technologies in educational contexts (e.g., Wu et al., 2024; Zheng et al., 2024). For example, Wu et al. (2024) employed TAM to examine the determinants of behavioral intention to use AI among 464 Chinese university-level EFL learners. Their findings revealed that AI attitude significantly and positively predicted behavioral intention to use AI, and that AI attitude exerted a significant positive mediating effect between perceived ease of use and students' behavioral intention to adopt AI technologies. Given the established importance of AI attitude in the behavioral intention to adopt AI technologies, in the present study, we chose generative AI attitude as another variable to explore its potential mediating effect in the relationships between PTS/GLM and learner well-being.

It has been posited that AI-integrated environments not only enhance linguistic outcomes but also establish “responsive and emotionally attuned learning ecosystems” that address learners' individual needs in dynamic and meaningful ways (Yang and Rui, 2025, p. 2). The Self-Determination Theory (Deci and Ryan, 2000; Ryan and Deci, 2000) suggests that humans' intrinsic motivation and well-being are fundamentally driven by three innate psychological needs: autonomy, competence, and relatedness. In the EFL educational contexts, when these needs of students are met, they are more likely to engage with the learning materials and develop positive emotional states (Yang and Rui, 2025). In effect, AI technologies can effectively support these needs through various mechanisms. To be specific, the personalized learning pathways offered by generative AI address students' autonomy needs by allowing learner-directed progression; the immediate adaptive feedback featured by generative AI satisfies students' competence needs by providing appropriately challenging yet achievable tasks; and the interactive communication tools, such as AI chatbots that simulate authentic social communication, help fulfill students' relatedness needs. This multi-faceted support system contributes substantially to the cultivation of EFL learners' intrinsic motivation and well-being.

Scholars have indeed observed that generative AI can enhance learner well-being. For instance, Linh et al. (2022) investigated the impact of AI chatbots on learner well-being, demonstrating that students who perceived supportive interactions with AI tools reported reduced negative learning experiences and increased satisfaction with the learning process. Huang et al. (2024) explored the roles of generative AI acceptance, perceived teachers' enthusiasm, and self-efficacy in predicting EFL learners' well-being. Their findings revealed that generative AI acceptance not only directly predicted EFL learners' well-being but also exerted an indirect influence on it through the mediating role of receptive skills self-efficacy. Additionally, Kohnke and Moorhouse (2025) explored how generative AI affects EFL learners' emotional engagement, motivation, and well-being, concluding that these technologies generally enhanced learning motivation, reduced anxiety and stress, and fostered an emotionally supportive learning environment.

However, the aforementioned research may have certain deficiencies. For instance, the well-being scale (i.e., the *Warwick-Edinburgh Mental Well-being Scale*) employed by Huang et al. (2024) is not targeted at EFL learning specifically, and therefore may not fully capture the domain-specific nature of well-being in EFL learning contexts (Li, 2025). The semi-structured interviews conducted by Kohnke and Moorhouse (2025) were not aimed at the construct of well-being, but were centered around the respondents' views on how generative AI affected the emotional dimension of language learning. Therefore, it can be considered that although the above research has deepened our understanding of learners' well-being in generative AI-integrated environments, there is still a lack of sufficient and effective empirical evidence on how generative AI affects learners' well-being in EFL learning contexts. The present study attempts to narrow the lacuna by targeting at L2 domain-specific well-being through refining and contextualizing Butler and Kern's (2016) *PERMA Profiler* to address EFL learners' well-being specifically.

### Mediating role of generative AI attitude in relationships among PTS, GLM, and learner well-being

Based on the literature review above and the identified gaps in existing research, this study aims to examine the potential direct predictive effects of PTS and GLM on learner well-being within AI-integrated learning environments, while investigating the possible mediating role of generative AI attitude in these relationships. To formulate hypotheses regarding the mediating effect of generative AI attitude, two prerequisite conditions must be met: (1) PTS/GLM exerts a significant influence on generative AI attitude, and (2) generative AI attitude significantly affects learner well-being. The second has been preliminarily addressed in the preceding section, with existing studies providing initial evidence supporting this significant relationship. Therefore, this section will focus on discussing the first prerequisite—namely, whether PTS or GLM significantly influences generative AI attitude.

To the best of our knowledge, although few study has directly explored the relationship between PTS and generative AI attitude [although a study along similar lines can be found in Wu et al. (2024)], it is probable that teacher support in the form of scaffolding, emotional support, respect and immediacy can serve as influential factors in promoting students' autonomy (Guo and Wang, 2025) and alleviating anxiety, fear, and stress regarding the use of AI technologies. Consequently, they may embrace AI technologies more readily than those without adequate PTS. Therefore, this study hypothesizes that PTS positively predicts generative AI attitude.

Moreover, in EFL learning, learners who adopt a GLM—believing that L2 proficiency can be improved through effort and is not constrained by the starting age of learning—are more inclined to seek challenging tasks (Zhang and Jiang, 2024), such as learning to utilize AI technologies to assist their EFL learning. Additionally, the higher the levels of learners' GLM, the more likely they are to experience positive emotions like enjoyment and pride (Wang H. et al., 2021) during EFL learning, as well as higher levels of self-efficacy (Wei and Chen, 2022). These positive emotions will aid in broadening their attentional scope, and fostering receptiveness to new learning (Fredrickson, 2001), thereby facilitating affective, behavioral, and cognitive acceptance of AI technologies. Thus, this study hypothesizes that GLM positively predicts generative AI attitude.

**Figure 1:**
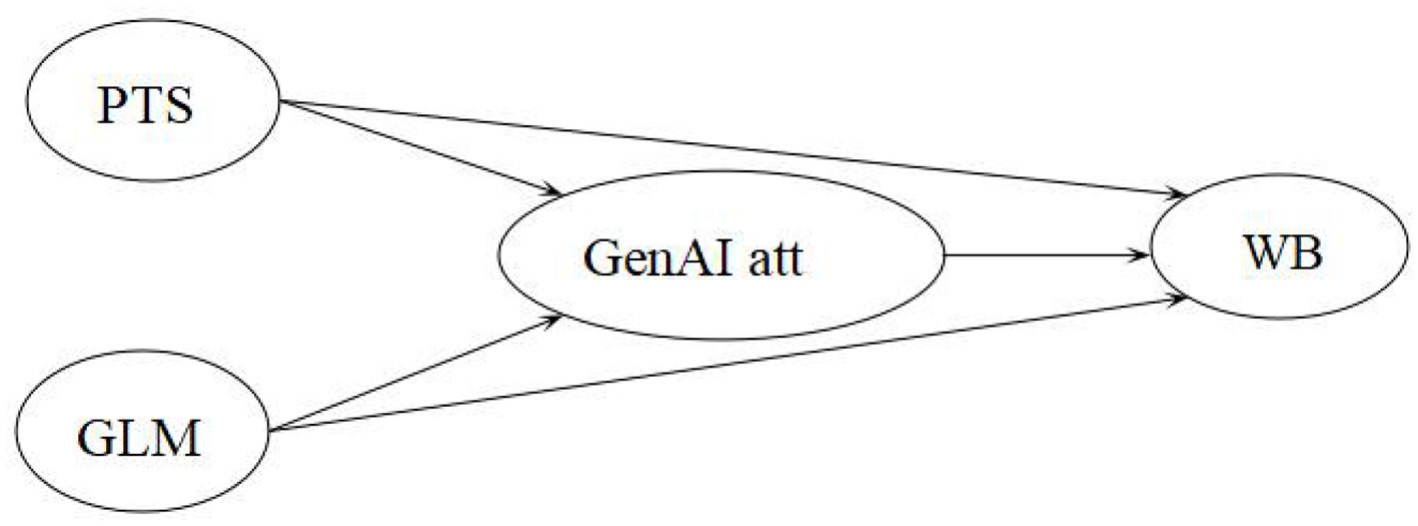
The hypothetical model in the present study. PTS, perceived teacher support; GLM, growth language mindset; GenAI att, generative AI attitude; WB, well-being.

Based on these theoretical assumptions and the preceding discussion on the relationships between PTS, GLM, generative AI attitude, and learner well-being, this study, focusing on Chinese EFL learners, proposes a hypothetical model (see Figure 1) and the following hypotheses:

H1: PTS positively predicts well-being.
H2: GLM positively predicts well-being.
H3: Generative AI attitude positively predicts well-being.
H4: Generative AI attitude plays a positive mediating role in the relationship between PTS/GLM and well-being.

## Method

### Participants

This study employed a convenience sampling method, recruiting 719 undergraduate students majoring in English-related specialties (including English, Business English, and Translation) across four academic years from eight universities in Jiangsu Province, China. The questionnaire was designed on Wenjuanxing, an online survey platform commonly used in China (https://www.wjx.cn/), which generated both a survey link and a QR code for dissemination via the QQ groups of the classes involved (QQ is a popular social networking software used in China for life and work). There were altogether 45 items on the questionnaire, including those collecting demographic information. Based on the criterion of Huang et al. (2012, p. 106), which recommends an average response time of at least 2 s per item for a questionnaire to be considered valid, this study excluded those questionnaires with completion time below 90 s, leaving 486 valid questionnaires for subsequent analysis, with an effective recovery rate of 67.60%. The respondents of these valid questionnaires were based in 7 cities in Jiangsu Province, including Taizhou (*N* = 208), Nanjing (*N* = 129), Nantong (*N* = 81), Suzhou (*N* = 41), Huai'an (*N* = 15), Xuzhou (*N* = 7), and Zhenjiang (*N* = 5), encompassing students from five first-tier universities (*N* = 194) and three second-tier undergraduate institutions (*N* = 292) in China.

The participants, aged 18 to 24 (M = 20.379, SD = 1.443), comprised 65 males (13.37%) and 421 females (86.63%). The distribution across academic years was as follows: 150 freshmen (30.86%), 124 sophomores (25.51%), 158 juniors (32.51%), and 54 seniors (11.11%). In terms of majors, 211 students (43.42%) were enrolled in English, 244 (50.21%) in Business English, and 31 (6.38%) in Translation. None of the participants had overseas study or living experience, and their experience of English learning ranged from 6 to 16 years (M = 12.167, SD = 2.097). Additionally, the participants reported 1 to 2 years of using generative AI, including ChatGPT, Deepseek, Wenxingyiyan, Doubao, Kimi, etc. in their everyday life and study.

### Research instruments

The study developed a composite questionnaire by directly adopting or refining existing scales (see below). To ensure participants' accurate comprehension, all the items were presented in Chinese. Among the scales used, the *GenAI-Assisted EFL Learning Inventory*, which measures learners' generative AI attitude, was originally developed in Chinese, and it was directly adopted in the current study. The *Students' Perceived EFL Teacher Support Scale*, which measures learners' perceived teacher support, was originally presented in a bilingual format (Chinese and English), and its Chinese version was directly adopted in the present study as well. The GLM scale, which measures learners' growth language mindset, was created by taking three items targeting GLM from *L2B* and *ASB* sub-scales, respectively, from *Language Mindsets Inventory*, and was originally presented in English. In this study, the technique of back translation was employed, following the procedures of forward translation, reconciliation, back translation, expert review, and pilot testing. Specifically, the two authors in this research independently translated the GLM scale into Chinese, and in cases of inconsistencies between them, they discussed about these and arrived at an agreed-upon version. Then, a doctorate in translation studies in the same school with the authors, who was not previously exposed to the original GLM scale, back translated it into English, and the authors then checked whether there were disparities between the translated and original version, and in cases of differences, modifications were made to the Chinese version. Subsequently, the GLM scale in Chinese, together with other scales used in this study, was subjected to an expert in applied linguistics for review and a further pilot testing with 30 students in the authors' university. These procedures were strictly followed to ensure conceptual equivalence, avoid cultural bias, and validate translation accuracy. Additionally, the *PERMA Profiler*, which originally targeted at everyday domains of life, was contextualized for EFL learning specifically, by adding expressions like “in English learning” and “learning English” to its items, and the same procedures of back translation were followed. The survey remained open for 5 days. Prior to data collection, researchers clearly explained the study's purpose and the intended use of the data to participants, and obtained their informed consent.

### PTS scale

This study employed the *Students' Perceived EFL Teacher Support Scale* developed by Liu and Li (2023), which was validated in the context of Chinese EFL learning. The instrument utilized a 5-point Likert scale comprising 12 items across three dimensions: academic support (5 items) (A sample item is “The English teacher shows us how to compensate for limited knowledge to solve some particular problems, such as guessing meanings of new words from the context, etc.”); instrumental support (3 items) (A sample item is “The English teacher shares online learning resources with me, such as word memorization software, etc.”); and emotional support (4 items) (A sample item is “The English teacher understands the difficulties in my English learning.”). The scale demonstrated excellent reliability in the current study, with a Cronbach's α coefficient of 0.957. Validity analysis yielded a KMO value of 0.951, and the Bartlett's test of sphericity was statistically significant (*p* < 0.001), with the rotated solution explaining 68.045% of the total variance. Confirmatory factor analysis (CFA) indicated satisfactory construct validity, with all fit indices meeting recommended thresholds: χ^2^/df = 3.420 < 5, CFI = 0.977 > 0.9, TLI = 0.970 > 0.9, RMSEA = 0.071 < 0.08, and SRMR = 0.027 < 0.08.

### GLM scale

The current study selected several items from the *Language Mindsets Inventory* originally developed and validated by Lou and Noels (2017). Specifically, we took 3 GLM-related items from each of two sub-scales—*Second Language Aptitude Beliefs (L2B)* and *Age Sensitivity Beliefs about Language Learning (ASB)*—to create a new 6-item scale. In previous studies, Wei and Chen (2022) combined *L2B* and *ASB* to form a new scale aimed at measuring Chinese EFL learners' fixed and growth language mindset, and its cultural adaptability of this scale was confirmed by demonstrating high reliability and validity. A sample item on *L2B* is “In learning a foreign language, if you work hard at it, you will always get better”, and that on *ASB* is “Regardless of the age at which they start, people can learn another language well”. The scale showed good reliability in the present study with a Cronbach's alpha of 0.913. The KMO value was 0.876 and the Bartlett's test of sphericity was statistically significant (*p* < 0.001), with the rotated solution explaining 70.073% of the total variance. The CFA results were acceptable: χ^2^/df = 4.658 < 5, CFI = 0.986 > 0.9, TLI = 0.973 > 0.9, RMSEA = 0.087, and SRMR = 0.022 < 0.08. While the RMSEA was slightly above the recommended 0.08 cutoff, the other fit indices were all within acceptable ranges.

### Generative AI attitude scale

The current study employed the *GenAI-Assisted EFL Learning Inventory* developed by Wang et al. (2024), which was validated in the context of Chinese EFL learning. This Likert 5-point scale consists of 15 items measuring three dimensions: affective (3 items, such as “I like to use generative AI to solve the problems I encounter in my English learning.”); behavioral (6 items, such as “I am willing to keep learning about how to use generative AI to assist in English learning.”); and cognitive (6 items, such as “I think generative AI is indispensable for English learners.”). Initial reliability analysis demonstrated excellent internal consistency (Cronbach's α = 0.943), with KMO value of 0.950 and a significant Bartlett's test of sphericity (*p* < .001). The cumulative variance explained after rotation reached 64.668%. However, CFA revealed sub-optimal construct validity (χ^2^/df = 6.170 > 5; CFI = 0.910; TLI = 0.892 < 0.9; RMSEA = 0.103 > 0.08; SRMR = 0.052). Consequently, two items with factor loadings below 0.7 (*GenAI-Behavior3* and *GenAI-Behavior4*) were removed from subsequent analysis. The revised scale maintained high reliability (Cronbach's α = 0.943) and validity (KMO = 0.952; Bartlett's test of sphericity: *p* < 0.001) and accounted for 59.910% of the total variance. CFA indicated acceptable model fit (χ^2^/df = 4.425 < 5; CFI = 0.951 > 0.9; TLI = 0.938 > 0.9; RMSEA = 0.084 ≈ 0.08; SRMR = 0.036 < 0.08), suggesting acceptable psychometric properties for research purposes. These results confirm that the modified scale demonstrates adequate reliability and validity for measuring attitudes toward generative AI in EFL contexts.

### Well-being scale

The well-being scale in this study was adapted from the first 15 items of the *PERMA Profiler* developed by Butler and Kern (2016), and validated in the context of Chinese EFL learning (Li, 2025). Since the original scale was not specifically designed to measure well-being in L2 learning, all the items in it were contextualized to focus exclusively on English learning. The modified Likert 5-point scale consists of 15 items assessing five dimensions: positive emotions, engagement, relationships, meaning, and accomplishment, with each dimension measured by three items. A sample item on the sub-scale of positive emotions is “I feel a great deal of contentment in my English learning”. One item on the sub-scale of engagement is “I am highly excited and interested in things, such as learning English”. An instance of the sub-scale items of relationships is “When I need it in my English learning, I receive help and support from others to a large extent”. An example item on the sub-scale of meaning is “I largely believe that what I do in my life is valuable and worthwhile, such as learning English”. One item on the sub-scale of accomplishment is “As for English learning, I often feel that I am making progress toward accomplishing my goals”.

Reliability analysis indicated excellent internal consistency (Cronbach's α = 0.970). The KMO value was 0.959, with the Bartlett's test of sphericity reaching statistical significance (*p* < 0.001), and the rotated solution cumulatively explained 70.354% of the total variance. CFA further supported the scale's construct validity, demonstrating an acceptable model fit (χ^2^/df = 4.211 < 5; CFI = 0.967 > 0.9; TLI = 0.957 > 0.9; RMSEA = 0.081 ≈ 0.08; SRMR = 0.026 < 0.08). These results confirm that the adapted scale is a reliable and valid instrument for assessing well-being in the context of EFL learning.

### Data analysis

The statistical analyses in this study were performed utilizing the SPSSAU platform (an online platform for performing statistical analysis at https://spssau.com). A series of analytical procedures were implemented, including descriptive statistics, normality tests, Pearson correlation analysis, regression analysis, and mediation analysis.

## Results

### Descriptive statistics and normality tests

The initial analysis involved conducting descriptive statistics and normality tests on the collected data, with the results presented in Table 1. As shown in Table 1, the skewness and kurtosis values for all variables fell within the range of ±1, indicating a normal distribution pattern. Furthermore, the mean scores for all variables were at or near the high-level range, suggesting generally favorable responses across measures.

**Table 1:** Descriptive statistics and normality tests of PTS, GLM, generative AI attitude, and well-being (*N* = 486).

**Variable**	**Mean**	**Min**.	**Max**.	**St. D**.	**Skewness**	**Kurtosis**
PTS	3.968	1.583	5.000	0.633	−0.055	−0.187
GLM	4.064	2.333	5.000	0.625	−0.107	−0.577
Generative AI attitude	3.982	2.385	5.000	0.567	0.056	−0.382
Well-being	3.818	1.133	5.000	0.642	0.034	0.269

PTS, perceived teacher support; GLM, growth language mindset; Std.D., standard deviation.

The Pearson correlation analysis (see Table 2) revealed significant positive correlations between PTS, GLM, generative AI attitude, and well-being, with strong correlation coefficients observed (*r* > 0.6). These results indicate that learners who perceive stronger teacher support, maintain a growth language mindset more strongly, and hold more positive attitudes toward generative AI tend to report higher levels of well-being.

**Table 2:** Correlations between PTS, GLM, generative AI attitude, and well-being (*N* = 486).

**Variable**	**1**	**2**	**3**	**4**
1. PTS	—			
2. GLM	0.638^**^	—		
3. Generative AI attitude	0.608^**^	0.542^**^	—	
4. Well-being	0.700^**^	0.663^**^	0.601^**^	—

PTS, perceived teacher support; GLM, growth language mindset.

** *p* < 0.01.

Furthermore, PTS demonstrated strong positive correlations with GLM, generative AI attitude, and well-being (*r* > 0.6). This suggests that EFL learners who perceive greater teacher support are more likely to develop a growth language mindset, exhibit more favorable attitudes toward generative AI, and experience enhanced well-being in their EFL learning.

Additionally, GLM showed significant positive correlations with generative AI attitude and well-being (*r* > 0.5). These findings imply that students who believe L2 proficiency can be improved through effort tend to hold more positive views of generative AI and report greater well-being in EFL learning contexts.

### Regression analysis and mediation effect test

Given the strong correlations observed among the independent variables (PTS, GLM), the mediating variable (generative AI attitude), and the dependent variable (well-being), it is necessary to examine potential multicollinearity among these variables. This study conducted linear regression analysis with well-being as the dependent variable and PTS, GLM, and generative AI attitude as independent variables. The results indicated variance inflation factor (VIF) values of 2.016, 1.801, and 1.694 for PTS, GLM, and generative AI attitude, respectively, all below the threshold of 5. This suggests a low likelihood of multicollinearity among these variables.

Subsequently, this study conducted a regression analysis on the hypothetical model, with PTS and GLM as independent variables, well-being as the dependent variable, generative AI attitude as the mediating variable, while controlling for gender, age, and years of English learning. As shown in Table 3, the analysis revealed three key findings: First, both PTS (β = 0.465, *p* < 0.001) and GLM (β = 0.366, *p* < 0.001) significantly and positively predicted well-being. Second, these two variables also significantly and positively predicted generative AI attitude (PTS: β = 0.461, *p* < 0.001; GLM: β = 0.248, *p* < 0.001). Finally, when all variables were included in the regression equation simultaneously, PTS (β = 0.370, *p* < 0.001), GLM (β = 0.314, *p* < 0.001), and generative AI attitude (β = 0.207, *p* < 0.001) all demonstrated significantly positive predictive effects on well-being.

**Table 3:** Results of the regression analysis on the relationships between variables in the mediation model.

**Regression equation**		**Overall fit indices**			**Significance of regression coefficient**	
**Outcome variable**	**Predictor variable**	**R** ^2^	**Adjusted R** ^2^	***F***	β	***t***
Well-being	Gender	0.571	0.566	127.704^***^	−0.042	−1.406
	Age				−0.007	−0.200
	Years of English learning				0.006	0.188
	PTS				0.465	11.880^***^
	GLM				0.366	9.371^***^
Generative AI attitude	Gender	0.428	0.422	71.738^***^	−0.023	−0.675
	Age				0.121	3.138^**^
	Years of English learning				0.021	0.543
	PTS				0.461	10.205^***^
	GLM				0.248	5.502^***^
Well-being	Gender	0.595	0.590	117.434^***^	−0.037	−1.280
	Age				−0.032	−0.966
	Years of English learning				0.002	0.060
	PTS				0.370	8.807^***^
	GLM				0.314	8.039^***^
	Generative AI attitude				0.207	5.379^***^

PTS, perceived teacher support; GLM, growth language mindset.

*** *p* < 0.001.

** *p* < 0.01.

Subsequently, this study employed the bias-corrected bootstrap method (with 5,000 resamples) to examine the potential mediating effect of generative AI attitude in the relationships between PTS/GLM and well-being (see Table 4). The results demonstrated two significant mediation pathways: First, PTS indirectly influenced well-being through generative AI attitude, with an effect size of 0.097 (accounting for 20.551% of the total effect); and the 95% confidence interval for this indirect effect did not straddle zero, indicating statistical significance. Second, GLM also indirectly influenced well-being through generative AI attitude, showing an effect size of 0.053 (representing 14.096% of the total effect), with the 95% confidence interval again excluding zero, indicating statistical significance.

**Table 4:** Results of bootstrap analysis of mediation effects.

**Pathway**	**c Total effect**	a^*^b Mediation effect	a^*^b (95% Boot CI)	**c' Direct effect**	**Conclusion**
PTS → attitude toward generative AI → Well-being	0.472	0.097	0.056 ~ 0.143	0.375	Partial mediation
GLM → attitude toward generative AI → Well-being	0.376	0.053	0.025 ~ 0.091	0.323	Partial mediation

## Discussion

### The direct predictive effect of PTS on well-being

The current study revealed that PTS significantly and positively predicted well-being, confirming Hypothesis 1 (H1). While limited research has explicitly investigated PTS as a predictor of well-being, the current finding allows for tentative comparisons with prior studies on PTS's influence on L2 emotions. For instance, Fang and Tang (2021) demonstrated that foreign language enjoyment among EFL learners was more strongly associated with their teachers and peers, while foreign language classroom anxiety was primarily linked to learners' emotions (such as fear of negative evaluation and speaking without preparation). The study by Zhao and Yang (2022) found that PTS indirectly influences learning engagement by positively influencing L2 students' enjoyment and negatively affecting their boredom. The research by Yang et al. (2024) revealed that teacher support is strongly correlated with grit (*r* = 0.647) and foreign language enjoyment (*r* = 0.671), and that teacher support significantly and positively predicts foreign language enjoyment (β = 0.277, *p* < 0.001).

The significant positive predictive effect of EFL students' PTS on their well-being can be reasonably explained through the theoretical framework of Self-Determination Theory (SDT) (Deci and Ryan, 2000; Ryan and Deci, 2000). According to SDT, the satisfaction of three basic psychological needs, i.e., autonomy, competence, and relatedness, serves as the fundamental driver of intrinsic motivation and well-being. When EFL students perceive that their teachers provide online and offline learning resources to them and dedicate extracurricular time to their English learning (instrumental support, satisfying autonomy and competence needs), help them acquire English language knowledge and skills while providing them with constructive feedback (academic support, satisfying competence needs), and demonstrate willingness to establish emotional connections with them (emotional support, satisfying relatedness needs), these fundamental psychological needs will be substantially satisfied, thereby contributing to the enhancement of their intrinsic motivation and well-being.

### The direct predictive effect of GLM on well-being

The present study demonstrated that GLM significantly and positively predicted well-being, thereby confirming Hypothesis 2 (H2). This finding aligns with prior research evidence on the effect of GLM on learner emotions. For instance, Wang H. et al. (2021) revealed that GLM positively predicted enjoyment and pride while negatively predicting boredom. Similarly, Wei and Chen (2022) found significant positive correlations between GLM and both enjoyment and self-efficacy, along with a negative correlation with anxiety, with GLM significantly predicting enjoyment. Zarrinabadi et al. (2022) established that GLM positively predicted enjoyment and negatively predicts anxiety, both through adaptability.

The significant positive predictive effect of GLM on well-being can be effectively interpreted through the lens of Achievement Goal Theory (Elliot and Church, 1997). This theoretical framework distinguishes three goal orientations: mastery goals (focusing on competence development and self-improvement) and performance goals (concerned with external evaluation and competitive ranking), which are further divided into performance-approach and performance-avoidance goals. EFL students with a GLM predominantly adopt mastery goals (e.g., Camacho et al., 2022; Lou and Noels, 2017), an orientation strongly associated with enhanced well-being through three key mechanisms.

First, GLM reduces performance anxiety and enhances learning engagement. Students with this mindset concentrate on mastery goals (e.g., “Today I will master this sentence pattern”), shifting their focus from social comparison to personal growth. This reorientation diminishes social-evaluative pressure and facilitates flow experiences—the state of complete absorption and enjoyment during language learning activities. Second, GLM strengthens resilience and facilitates adaptive failure attribution. When encountering setbacks in language learning (e.g., exam failures or communication breakdowns), students with GLM demonstrate greater resilience. They attribute failures to controllable factors (e.g., “I need more listening practice”) rather than fixed abilities (e.g., “I lack language talent”) (Zhang and Jiang, 2024). This adaptive attribution reduces helplessness and sustains long-term motivation, which is expected to contribute to enhanced levels of well-being. Third, GLM promotes positive academic emotions. According to Pekrun's (2006) Control-Value Theory, EFL students will experience heightened positive emotions (e.g., hope and pride) when they perceive control over the learning process and outcome and perceive high value of the learning content. In actuality, EFL students with GLM inherently possess these two cognitive characteristics. By definition, EFL students with a GLM believe their English skills can be improved through effort. As a result, they feel more in control of their learning process and outcomes. In China's EFL context—where universities, parents, and students all place high importance on English education—they also tend to view EFL learning as highly valuable and useful. Consequently, they are likely to experience more positive emotions from the learning process, ultimately enhancing their overall well-being.

### The direct predictive effect of generative AI attitude on well-being

The current study found that generative AI attitude significantly and positively predicted well-being, thus confirming Hypothesis 3 (H3). This result corroborates existing research findings. For instance, Linh et al. (2022)) demonstrated that AI chatbots reduced students' negative learning experiences while enhancing their satisfaction with the learning process. Similarly, Huang et al. (2024) revealed that generative AI acceptance positively predicted EFL learners' well-being. Kohnke and Moorhouse (2025) substantiated that generative AI generally enhanced students' learning motivation, reduced anxiety and stress, and fostered an emotionally supportive learning environment. Zong and Yang (2025) uncovered that AI-enhanced social-emotional learning significantly fostered students' engagement and enhanced their emotional well-being.

The significant positive prediction of EFL students' well-being by their generative AI attitude can be reasonably explained through the perspective of Broaden-and-Build Theory (Fredrickson, 2001). This theoretical framework posits that positive emotions broaden individuals' attention and mind while expanding their behavioral repertoires. When accumulated over time, these short-term effects facilitate the construction of enduring physiological, psychological, and social resources that promote personal development, which in turn generates further positive emotions. Additionally, the theory maintains that positive emotions can counteract the detrimental effects of negative emotions, thereby cultivating resilience and well-being (Li et al., 2024).

Consequently, when EFL learners hold positive attitudes toward generative AI—such as believing that generative AI tools are indispensable for EFL learning, finding generative AI tools particularly helpful for addressing challenges in EFL learning, and being willing to try using generative AI to assist EFL learning—they would exhibit significant behavioral changes and improved psycho-emotional states. This enhanced psychological condition elevates well-being through multiple dimensions.

From an affective perspective, EFL learners with favorable generative AI attitudes typically demonstrate stronger motivation for technological engagement and lower technology-related anxiety, stress, and fear. By perceiving generative AI as collaborative partners rather than evaluators, these learners more readily experience enjoyment and accomplishment during its usage, which helps to elevate levels of well-being among them. Cognitively, EFL learners with positive attitudes toward generative AI tend to conceptualize it as intelligent scaffolding, proactively utilizing it for language practice, and reflective learning. Through this process, they recognize the multifaceted benefits of generative AI for EFL learning, developing stronger perceptions of the technology's usefulness and ease of use, which subsequently enhances their well-being. Behaviorally, positive attitudes toward generative AI may well translate into more frequent and profound engagement with generative AI tools or systems. This deeper interaction enables learners to more fully experience how these technologies satisfy their basic psychological needs, ultimately generating higher levels of intrinsic motivation and well-being.

### The mediation effect of generative AI attitude

This study found that generative AI attitude plays a partial and positive mediating role in the relationship between PTS/GLM and well-being, confirming Hypothesis 4 (H4). In other words, PTS and GLM not only directly predict well-being but also indirectly influence it through the mediating effect of generative AI attitude.

First, PTS positively predicts generative AI attitude, which in turn positively predicts well-being. This suggests that learners who perceive academic, instrumental, and emotional support from EFL teachers tend to hold more positive attitudes toward generative AI. As mentioned earlier, while the introduction of new technologies such as generative AI may bring novelty to its use, learners might also experience discomfort, anxiety, or frustration when using these tools, especially when unfamiliar with them or receive inadequate support. Timely and sufficient support from EFL teachers can help mitigate these negative emotions. Specifically, when teachers effectively demonstrate how to use generative AI for EFL learning, students become more willing to try these tools. As learners gain proficiency in using generative AI, they increasingly recognize its powerful supportive role in EFL learning and perceive their progress in EFL leaning over time, which gradually enhances their sense of well-being in the learning process. From a theoretical perspective, the Broaden-and-Build Theory posits that positive emotions broaden one's attention and mind, build personal resources and facilitate receptiveness to new learning. Through providing academic, instrumental and emotional support, EFL instructors will engender more positive emotions in learners, and this will facilitate their exploration of new technologies such as generative AI, particularly when they encourage and guide them in the use of generative AI tools or systems. Under these circumstances, EFL teachers will alleviate students' technological anxiety and stimulate their willingness to experiment with these tools or systems.

Second, GLM positively predicts generative AI attitude, which in turn positively predicts well-being. This result indicates that learners with a GLM are more receptive to generative AI. As previously discussed, GLM refers to the belief that language abilities and L2 aptitude are malleable. EFL learners with this mindset believe their English proficiency can be improved through sustained effort, motivating them to actively engage in the learning process. They proactively seek resources, such as study materials, online resources, and efficiency-enhancing softwares (including generative AI), to support their learning. This may explain why GLM learners are more open to adopting generative AI. From the perspective of Achievement Goal Theory, learners with a growth mindset are more inclined to adopt mastery goals (e.g., Camacho et al., 2022; Lou and Noels, 2017), prioritizing skills and competence development over performance outcomes and external evaluations. This orientation, along with their initial experience in using generative AI (such as Deepseek and Doubao), may lead them to view generative AI as a helpful learning tool, actively leveraging the affordances of generative AI in assisting EFL learning. Essentially, GLM cultivates more positive attitudes toward technology adoption by intrinsically linking AI usage with self-development.

## Educational implications

One of the findings of this study is that PTS directly predicts EFL learners' well-being in AI-integrated environments, highlighting the importance of PTS in enhancing well-being among EFL learners. In an era of rapid development of generative AI technologies, EFL teaching and learning are undergoing transformative changes. Learners experience both the conveniences brought by technological updates—which generate positive emotions like joy, hope, and pride in English learning—as well as potential uncertainty and maladaptation that may lead to negative emotions such as anxiety, stress, and frustration. Consequently, EFL teachers need to provide comprehensive support, particularly in instructing learners how to utilize generative AI tools or systems to facilitate English learning. For instance, beyond imparting English language knowledge and training students' language skills, EFL instructors should also educate learners about fundamental knowledge of generative AI, introduce commonly used AI tools or systems, and demonstrate their applications in EFL learning. This approach is likely to enhance learners' AI literacy while mitigating the negative emotions evoked in its use and subsequently increasing their acceptance of generative AI.

Another finding of this study is that GLM significantly and positively influences EFL learners' well-being, underscoring the pivotal role of GLM in elevating learner well-being in AI-integrated environments. It is suggested by researchers that GLM not only directly affects students' learning behaviors but also impacts various emotions experienced during the learning process (King et al., 2012; Lou and Noels, 2016), thereby influencing their perceived well-being in EFL learning. It is therefore essential for EFL teachers to conduct reliable and valid surveys to assess learners' GLM levels, implement targeted interventions (e.g., Rong and Liu, 2024) for students with lower GLM to improve their GLM. Given the domain-specific nature of language mindset (e.g., Rong and Liu, 2024), we recommend teachers develop skill-specific interventions (i.e., listening, speaking, reading, writing) to promote more active engagement in English learning, foster positive L2 emotions, and ultimately enhance both learning outcomes and student well-being.

Lastly, this study uncovered that generative AI attitude directly predicted well-being and concurrently mediated the relationship between PTS/GLM and well-being. Therefore, the attitudes toward generative AI play a crucial role in enhancing learner well-being. To foster more positive attitudes among learners, the following strategies can be taken into account. Firstly, EFL teachers are recommended to monitor students' progress in learning to use generative AI, promptly address their difficulties and confusions, build empathy with them, and establish a learning community where teachers and students collaboratively advance their proficiency in utilizing generative AI. Secondly, EFL instructors are advised to improve their students' emotional intelligence (EI) to enhance their abilities in perceiving, understanding, using, and regulating their own emotions in the process of learning to use generative AI, as research shows that EI has the potential to enhance students' psychological adjustment, emotional management, happiness, optimism, well-being, among others (Yang and Duan, 2023). Additionally, EFL teachers should endeavor to cultivate a positive classroom climate to foster a psychologically safer, more motivating and engaging environment for students to practice using generative AI by enhancing class mutual respect and devoting more attention to teacher-student and student-student interactions (Yang and Lin, 2024).

For EFL learners, the findings of this study also carry profound implications. This study revealed that perceived teacher support directly enhanced EFL learners' well-being in AI-integrated environments. This finding suggests that when encountering challenges posed by AI technologies, learners should proactively seek guidance and support from teachers. Specifically, when facing confusion or difficulties in adapting to AI tools (e.g., ChatGPT, Deepseek), learners should promptly communicate their concerns to their instructors rather than enduring anxiety or frustration in isolation. Teachers may provide training on the fundamental principles, appropriate applications, and potential limitations of AI tools. Learners should actively engage in such training to improve their AI literacy and mitigate negative emotional responses to technology use. Additionally, forming AI study groups with peers to share experiences and foster mutual assistance can strengthen their adaptive capabilities.

The research further indicated that a growth language mindset significantly improved learners' well-being. This finding yields several important implications for EFL learners. First, learners should abandon fixed mindset such as “I'm naturally bad at English” or “AI outperforms me regardless of my effort,” and instead cultivate the conviction that consistent practice and proper strategies lead to improvement in EFL learning. Second, learners can establish short-term achievable goals, such as practicing spoken English with AI chatbots for 10 min daily or using AI to revise one composition weekly. The accomplishment of these incremental objectives helps build a sense of accomplishment, which is an important element of well-being. Moreover, learners should focus on progress rather than perfection. While AI provides immediate feedback, learners need not pursue flawless performance but rather appreciate their developmental gains to reduce their anxiety.

The study also demonstrated that attitudes toward generative AI not only directly influenced well-being but also mediated the relationships between PTS/GLM and well-being. Consequently, developing appropriate perspectives on AI is crucial. First, learners should maintain a balanced view of AI's role by neither over-relying on it nor rejecting it due to fears of replacement. Instead, they should utilize AI as a supplementary tool for tasks like grammar checking, vocabulary expansion, or conversational practice. Second, learners should participate in positive AI-enabled learning environments where instructors cultivate respectful and interactive classroom atmosphere. Through guided uses with AI tools in such settings, learners can reduce technophobia and develop constructive attitudes toward generative AI, thereby enhancing learning-related well-being.

## Conclusion

This study examined the relationships among perceived teacher support, growth language mindset, generative AI attitude, and well-being in AI-integrated environments, with a sample of 486 Chinese university EFL learners. The results demonstrated that perceived teacher support, growth language mindset, and generative AI attitude all significantly and positively predicted well-being. Furthermore, generative AI attitude partially mediated the relationships between perceived teacher support/growth language mindset and well-being. These findings shed light on the influential factors and underlying mechanisms of learner well-being in AI-integrated environments, underscoring the crucial roles of perceived teacher support and growth language mindset in shaping learners' attitudes toward generative AI and enhancing their well-being. Theoretically, the findings of this study helps advance our understanding of the intricate interplay among personal, technological and contextual factors and their combined influence on learning well-being within AI-integrated environments, and the proposed model may trigger more studies along similar lines in future research endeavors. Practically, the educational implications gleaned from these findings may be instrumental in assisting EFL teacher in providing more appropriate teacher support, fostering higher levels of growth language mindset in their students, cultivating more positive attitudes toward generative AI, and ultimately enhancing their learners' well-being.

Despite these preliminary findings, several limitations should be acknowledged. First, the study exhibited a gender imbalance, with female participants constituting the majority (86.63%), which may impact the generalizability of the findings, particularly regarding the emotional experiences and learning outcomes of EFL learners. It is recommended that future studies aim for a more balanced gender distribution to improve the external validity of the findings. Second, the perceived teacher support scale used in this study was designed for general EFL learning contexts rather than specifically for AI-integrated environments. Given that perceived teacher support may be context-dependent, future studies should adapt the scale to better capture teacher support in AI-enhanced learning settings. Third, structural equation modeling (SEM) was not employed in this study due to insufficient quality of the data collected resulting in sub-optimal fit indices of the structural model. It is recommended that future studies improve the sample size and the quality of the data and try using SEM, as it is well-suited for handling complex relationships between multiple latent variables, providing more precise mediation estimates and assessing both direct and indirect effects, which would enhance the interpretability and credibility of the findings. Fourth, this study did not incorporate semi-structured interviews with learners, missing an opportunity to triangulate data and explore the deeper reasons behind the observed relationships among variables. Adopting a mixed-methods approach in future research could provide more comprehensive and nuanced insights into the factors influencing learner well-being and their mechanisms in AI-integrated environments. Lastly, the cross-sectional nature of this study may have limitations in deriving causal relationships between the variables under study. It is suggested that future studies employ research approaches such as latent growth curve analysis to explore the complex and dynamic interplay among perceived teacher support, growth language mindset, generative AI attitude, and well-being.

## Data Availability

The original contributions presented in the study are included in the article/Supplementary material, further inquiries can be directed to the corresponding author.

## Supplementary material

SUPPLEMENTARY TABLE S1 Questionnaires for generative AI attitude, perceived teacher support, growth language mindset, and learner well-being.

SUPPLEMENTARY TABLE S2 Data collected and used in this study.
