# Supplementary material for: The effects of perceived teacher support and growth language mindset on learner well-being in AI-integrated environment: the mediating role of generative AI attitude
Source: Front Psychol. 2025 Sep 16;16:1660462. doi: 10.3389/fpsyg.2025.1660462 (PMC12481513; doi:10.3389/fpsyg.2025.1660462)
Supplement: SUPPLEMENTARY TABLE S1 — Questionnaires for generative AI attitude, perceived teacher support, growth language mindset, and learner well-being. [file Table_1.docx]

生成式AI态度、感知教师支持、成长型语言思维模式、学习者幸福感关系

调查问卷

说明：

性别：

年龄：

学习英语年限：

专业：

年级：

Q1: 我感觉使用生成式AI辅助学习英语的过程非常有趣；

Q2: 我喜欢借助生成式AI来解决我在英语学习中遇到的问题；

Q3: 生成式AI辅助英语学习让我感到自信，觉得学习英语更容易了；

说明：情感维度

Q4: 我愿意尝试使用生成式AI辅助英语学习；

Q5: 我想尝试使用生成式AI进行个性化的英语学习；

Q6: 如果学校开设有关生成式AI辅助英语学习的课程，我会参加；

Q7: 如果有一个与生成式AI辅助英语学习相关的社团，我想加入；

Q8: 我愿意持续学习有关如何使用生成式AI辅助英语学习的知识；

Q9: 我想探索一些生成式AI使用方法，让英语学习更加便捷；

说明：行为维度

Q10: 我认为未来的英语学习将与生成式AI密切结合；

Q11: 我认为每个学生都应该掌握生成式AI辅助英语学习的方法；

Q12: 我认为生成式AI对英语学习者是不可或缺的；

Q13: 我认为值得学习使用生成式AI来提高英语水平；

Q14: 我认为我可以有效地利用生成式AI来辅助我的英语学习；

Q15: 我认为生成式AI在英语学习中带来的益处大于弊端；

说明：认知维度

Q16: 英语老师针对我们英语学习中的薄弱点进行专项教学（如语法点）；

Q17: 英语老师教我们如何在现有知识不足的情况下解决一些特定问题（如通过上下文推测生词词意等）；

Q18: 英语老师教我们词汇的搭配模式以及篇章结构等语言知识；

Q19: 英语老师给我们拓展与课本内容相关的课外文化背景知识；

Q20: 英语老师教我们 “干货” 知识（如写作句型等）；

说明：学业支持

Q21: 英语老师帮我选择合适的教辅资料；

Q22: 英语老师帮我选择合适的课外读物；

Q23: 英语老师给我分享在线学习资源（如背单词软件等）；

说明：工具支持

Q24: 英语老师很关心我的学习情况；

Q25: 英语老师对我很有耐心，即便我的基础再差也不放弃我；

Q26: 英语老师对我有很高的期望；

Q27: 英语老师理解我学习英语的困难之处；

说明：情感支持

Q28: 你的外语能力总是可以提升的；

Q29: 学习外语时，只要努力就一定会进步；

Q30: 如果真正下功夫，你的外语使用水平总会不断提高；

说明：Second language aptitude beliefs (L2B)

Q31: 无论年龄大小，只要肯努力，人人都能学好外语；

Q32: 一个人外语学得好不好与年龄无关，勤奋学习的人都可以流利地说这门外语；

Q33: 无论从什么年龄开始学习，人们都能掌握好一门新语言；

说明：Age sensitivity beliefs about language learning (ASB)

Q34: 一般来说，我在英语学习中常常感到快乐；

Q35: 一般来说，我在英语学习中常常有积极的情绪；

Q36: 一般来说，我在英语学习中感受到很大程度的满足；

说明：积极情绪

Q37: 我常常沉浸于自己正在做的事情中（如英语学习）；

Q38: 一般来说，我对各种事情感到兴奋和兴趣的程度很高（如英语学习）；

Q39: 在做自己喜欢的事情时，我常常会忘却时间（如英语学习）；

说明：投入

Q40: 当我在英语学习中有需要时，我在很大程度上能够得到他人的帮助与支持；

Q41: 在英语学习中，我在很大程度上感受到他人的关爱；

Q42: 我对自己在英语学习活动中建立的人际关系感到很满意；

说明：关系

Q43: 一般来说，学习英语让我感觉在很大程度上过着一种有目的、有意义的生活；

Q44: 一般来说，我在很大程度上认为我在生活中所做的事情有价值、值得做（如英语学习）；

Q45: 学习英语让我在很大程度上认为我在生活中有方向感；

说明：意义

Q46: 就英语学习来说，我常常感到在实现自己的目标方面正在取得进步；

Q47: 就英语学习来说，我常常能够实现自己设定的重要目标；

Q48: 就英语学习来说，我常常可以处理好自己的各项任务。

说明：成就
